# Infection of *Galleria mellonella* (Lepidoptera) Larvae With the Entomopathogenic Fungus *Conidiobolus coronatus* (Entomophthorales) Induces Apoptosis of Hemocytes and Affects the Concentration of Eicosanoids in the Hemolymph

**DOI:** 10.3389/fphys.2021.774086

**Published:** 2022-01-06

**Authors:** Anna Katarzyna Wrońska, Agata Kaczmarek, Michalina Kazek, Mieczysława Irena Boguś

**Affiliations:** ^1^Witold Stefański Institute of Parasitology, Polish Academy of Sciences, Warsaw, Poland; ^2^BIOMIBO, Warsaw, Poland

**Keywords:** *Galleria mellonella*, *Conidiobolus coronatus*, eicosanoids, apoptosis, necrosis, insects hemocytes

## Abstract

Apoptosis and autophagy, the mechanisms of programmed cell death, play critical roles in physiological and pathological processes in both vertebrates and invertebrates. Apoptosis is also known to play an important role in the immune response, particularly in the context of entomopathogenic infection. Of the factors influencing the apoptotic process during infection, two of the lesser known groups are caspases and eicosanoids. The aim of this study was to determine whether infection by the entomopathogenic soil fungus *Conidiobolus coronatus* is associated with apoptosis and changes in caspase activity in the hemocytes of *Galleria mellonella* larvae, and to confirm whether fungal infection may affect eicosanoid levels in the host. Larvae were exposed for 24 h to fully grown and sporulating fungus. Hemolymph was collected either immediately after termination of exposure (F24 group) or 24 h later (F48 group). Apoptosis/necrosis tests were performed in hemocytes using fluorescence microscopy and flow cytometry, while ELISA tests were used to measure eicosanoid levels. Apoptosis and necrosis occurred to the same degree in F24, but necrosis predominated in F48. Fungal infection resulted in caspase activation, increased PGE1, PGE2, PGA1, PGF2α, and 8-iso-PGF2α levels and decreased TXB2 levels, but had no effect on TXA2 or 11-dehydro-TXB2 concentrations. In addition, infected larvae demonstrated significantly increased PLA2 activity, known to be involved in eicosanoid biosynthesis. Our findings indicate that fungal infection simultaneously induces apoptosis in insects and stimulates general caspase activity, and this may be correlated with changes in the concentrations of eicosanoids.

## Introduction

Like cell proliferation and cell differentiation, programmed cell death also has a crucial role in biological growth and development. Two primary programmed cell death signaling pathways are known: apoptosis and autophagy. Both are evolutionarily conserved and can be induced by a range of endogenous and exogenous factors, including the levels of toxins, hormones, growth factors, nitric oxide and cytokines, as well as by heat, irradiation, nutrient deprivation, infection, hypoxia and increased intracellular calcium concentration (Elmore, 2007; Kazek et al., 2020; Cao et al., 2021; Nury et al., 2021; Wang et al., 2021). In addition, the apoptotic pathway has characteristic morphological changes and biochemical processes, including cytoplasmic and nuclear condensation, phosphatidylserine extrusion, vacuolization, chromatin condensation and DNA fragmentation; in addition, a number of apoptotic bodies are formed that are ultimately engulfed by surrounding cells (D’Arcy, 2019).

While autophagy involves cell degradation by internal lysozymes (Khandia et al., 2019), cell death can also occur by necrosis: a passive process that does not require energy input. During necrosis, groups of cells typically swell and lose the continuity of their cell membranes due to the expression of harmful factors; however, unlike apoptosis, the breakdown of organelles and the cell as whole is accompanied by inflammatory reactions (Zhang et al., 2020).

Two main apoptotic pathways are believed to exist: the extrinsic/receptor pathway, associated with the cell membrane, and the intrinsic/mitochondrial pathway, involving the mitochondria (Bedoui et al., 2020). Both pathways have been widely described in mammals. However, an additional pathway involving T-cell mediated cytotoxicity and perforin-granzyme-dependent killing of the cell has also been proposed, which can induce apoptosis *via* either granzyme B or granzyme A (van Daalen et al., 2020). Each pathway is activated by a different initiator caspase: caspase-8 for the extrinsic pathway, caspases-9 and -2 for the intrinsic pathway, and caspase-10 for the granzyme B pathway. These in turn activate executioner caspases 3, 6, or 7. However, the granzyme A pathway works in a caspase-independent fashion.

Caspases such as cysteine-aspartic proteases, cysteine aspartases or cysteine-dependent aspartate-directed proteases have proteolytic activity; however, they are typically expressed in an inactive proenzyme form. Caspases can be divided into three groups based on the structure of the protease domain: one group, comprising caspases 1, 4, 5, 11, 12, 13, and 14, are known to have a pro-inflammatory effect that activates cytokines, a second group consisting of caspases 9, 8, 2, and 10 are believed to act as initiator caspases, while a third group comprising caspases 3, 6, and 7 act as executive (effector) caspases (Bock and Tait, 2020; Kesavardhana et al., 2020). The target enzymes enhance the degradation of a range of key cellular proteins, including those involved in RNA synthesis and splicing, cell adhesion and cell cycle management; they also play roles in various aspects of DNA expression, i.e., synthesis, cutting, repair, binding and transcription; they also influence protein G-mediated signaling, as well as protein synthesis, degradation and modification, as well as calcium metabolism (Nirmala and Lopus, 2020).

Although apoptosis is known to occur in various groups of organisms, the process is not well understood in insects and is believed to act through different processes than in mammals. Most relevant articles examine apoptosis in holometabolous insects, whose development is characterized by a complete metamorphosis between a wingless larval stage, mostly dedicated to nutrient acquisition and growth, and a winged adult form dedicated to reproduction (Buszczak and Segraves, 2000; Romanelli et al., 2016). These processes have been well described in the literature in insects of the order Lepidoptera (Lockshin and Zakeri, 1994; Uwo et al., 2002; Parthasarathy and Palli, 2007; Tettamanti and Casartelli, 2019).

Among all insects studied so far, the mechanisms of apoptosis have been best described in *Drosophila* spp. (Zhou, 2019; Kidera et al., 2020; Li et al., 2020). In this genus, seven apoptotic caspases have been identified, including the DCP-2/DREDD protein, a caspase-8 homolog whose role in apoptosis is unknown, the DRONC protein (major caspase, caspase-9 homolog) and DCP-1 (Death caspase protein), as well as the executive caspases DrICE (*Drosophila* interleukin converting enzyme), DECAY (Death executioner caspase) and Damm (Death associated molecule related to Mch2 caspase). In addition, DARK (*Drosophila* Apaf-1 related killer) is believed to be homologous to the Apaf-1 (Apoptotic protease activating factor 1) protein in mammals (McSharry and Beitel, 2019).

Although, the precise nature of the receptors mediating apoptosis in *Drosophila* spp. remains under discussion. A FADD homolog (Fas-associated protein with death domain) has been isolated and it has been shown to work in cooperation with the initiator DREDD caspase.

Furthermore, the Eiger protein has been found to induce apoptosis independently of DREDD (caspase 8 homolog) in *Drosophila* spp., despite it being a homolog of mammalian TNF (tumor necrosis factor) (Igney and Krammer, 2002; Li et al., 2019). Activation of programmed cell death in *Drosophila* is mainly dependent on three proapoptotic proteins: RPR (reaper), GRIM and HID (head involution defective) which are widely described in the literature (Hay and Guo, 2006; Abdelwahid et al., 2011; Tettamanti and Casartelli, 2019).

In contrast, very little is known about apoptotic molecular pathways in the Lepidoptera (Ma et al., 2021). The discovery in the early 1990s of p35, a baculovirus pan-caspase inhibitor (Clem et al., 1991), led to the characterization of the first lepidopteran caspase, Sf-caspase-1, from a Sf9 cell line, derived from *Spodoptera frugiperda* ovary cells (Ahmad et al., 1997). This protein is similar to the caspase-3 of mammals and DrICE in *Drosophila* spp. Its crystal structure is also similar to those of human caspases 1, 3, 7, 8, and 9, the only difference being the orientation of the amino terminus of the large subunit (Forsyth et al., 2004).

Other analogs to this caspase have been identified in *Spodoptera littoralis* (Sl-caspase-1), *Spodoptera litura* (Sl-Apaf-1), *Helicoverpa armiger* (Hearm caspase-1), and *Trichloplusia ni* (Accorsi et al., 2015; Ma et al., 2021). In the silkworm *Bombyx mori*, two initiator caspase genes (Nedd2-like 1 and Nedd2-like 2) and Bm-caspase-1have been described (Tian et al., 2012; Wang et al., 2017). In 2012, Gm-caspase-1 was also characterized in *G. mellonella*, which is becoming an increasingly popular model in innate immunity and toxicological studies (Wojda et al., 2020). Its sequence is 75% identical to Sl-1 caspase, and 41% identical to human caspase-3, and the protein is believed to be mainly involved in the metamorphosis process (Khoa et al., 2012; Poyraz Tinartas et al., 2021).

Apoptosis is becoming recognized as a fundamental component of the immune system (Brokatzky et al., 2019; Quarleri et al., 2021; Riera Romo, 2021). It is involved in a range of cellular processes; however, these fall into two broad categories: the development and shaping of the immune receptor repertoire, and immune effector mechanisms. Although its effects on the immune system have been primarily observed during viral infections (Feig and Peter, 2007), they are also believed to play a key role during fungal infection (Allen and DeepeJr., 2005).

Due to their similarity with the immune mechanisms of mammals, the immune system of insects has been described as exemplifying “the roots of human innate immunity” (Sheehan et al., 2018). In *G. mellonella*, innate immunity comprises cellular and humoral immunity. In these insects, phagocytosis, encapsulation and nodulation typically involve granulocytes and plasmatocytes with adherent properties, while non-adhesive spherule cells transport cuticle components, and oenocytoids carry phenoloxidase precursors (Lavine and Strand, 2002; Eleftherianos et al., 2021). However, humoral immunity is constitutive, and infection-induced synthesis of defense molecules such as radicals, proteins and antimicrobial peptides (AMPs; in *G. mellonella*: cecropins, gallerimycin, galiomicin, and moricin-like peptides) can be observed, as well as melanization and hemolymph clotting (Cytrynska et al., 2007; Nguyen et al., 2011; Wojda, 2017). In insects, the immune response is known to be regulated by three major pathways, *viz.* Toll, Imd and JAK-STAT, which are analogous to certain pathways in the human immune system (Marmaras and Lampropoulou, 2009; Zhang et al., 2021); however, other pathways that could regulate insect immunity are increasingly being investigated.

One group of compounds that have been extensively studied in mammalian systems for their roles in immune regulation and inflammation are the eicosanoids. These are known to act by modulating cytokine release, cell differentiation, cell migration, antigen presentation and apoptosis (Dennis and Norris, 2015). Similar conserved responses have also been described in insects, with eicosanoids being known to mediate cellular immune function through phagocytosis, encapsulation or melanization (Park and Kim, 2000; Shrestha and Kim, 2008; Stanley et al., 2012; Al Baki et al., 2021).

In mammals eicosanoid biosynthesis is believed to be promoted by the activity of phospholipase A2 (PLA2), which cleaves fatty acids to release arachidonic acid (AA). The released AA is used to synthesize eicosanoids *via* three pathways: the cyclooxygenase (COXs) pathway, which generate prostaglandins (PGs) and thromboxanes (TXs); the lipoxygenase (LOX) pathway, which enables the production of leukotrienes (LTs), lipoxins (LXs) and hydroxyeicosatetraenoic acids (HETEs) and hydroperoxyeicosatetraenoic acids (HPETEs); as well as the cytochrome P450 pathway, which enables the generation of PGs, TXs, HETEs, HPETEs and epoxyeicosatrienoic acids (EETEs) (Khanapure et al., 2007; Haeggstrom and Funk, 2011).

The process of eicosanoid biosynthesis and degradation in insects has been described in detail by Kim and Stanley (2021). Briefly, phospholipase A2 (PLA2) catalyzes the hydrolysis of linoleic acid (LA) from membrane-associated phospholipids (PLs); the resulting LA is elongated by long-chain fatty acid elongase (Elo) and then desaturated by desaturase (Des) to arachidonic acid (AA). AA is then oxygenated by epoxidase (EPX) into epoxyeicosatrienoic acid (EET), lipoxygenase (LOX) into leukotriene (LT), or cyclooxygenase-like peroxynectin (Pxt) to prostaglandin (PG). The EETs are degraded by soluble epoxide hydrolase (sEH). LTA4 is formed from 5-hydroxyperoxide eicosatetraenoic acid (HpETE) and changed into LTB4 by LTA4 hydrolase (LTA4H) or into LTC4 by glutathione peroxidase (Gpx). Finally, various PGs are formed from PGH2 by cell-specific enzymes, thromboxane A2 (TXA2) synthase (TXAS), PGD2 synthase (PGDS), PGE2 synthase (PGES), and PGI2 synthase (PGIS); these PGs are degraded by PG dehydrogenase (PGDH) and PG reductase (Kim and Stanley, 2021).

However, although the role of eicosanoids in apoptosis is very interesting, our knowledge of their activity remains incomplete. While the apoptosis-regulating roles of eicosanoids generated through the LOX, COX, and P450 pathways have been studied extensively in mammals in the context of tumorigenesis and cancer, these eicosanoids also participate in the apoptosis of non-cancer tissues. In both cases, they are known to act *via* the extrinsic and intrinsic apoptotic pathways. Interestingly, eicosanoids appear to possess both pro- and anti-apoptotic functions depending on their cellular location (i.e., intracellular *vs.* extracellular), differentiation stage and cell tissue origin (Funk, 2001; Yin et al., 2013).

As apoptosis has been demonstrated to be one of the key processes in the immune response cascade (Nagata and Tanaka, 2017) the following hypothesis was formulated: fungal infection stimulates apoptosis and influences eicosanoid levels in immunocompetent insect hemocytes.

Based on this hypothesis the aim of the present study was to determine whether infection with the entomopathogenic soil fungus *C. coronatus* influences apoptosis in *G. mellonella* hemocytes and changes in caspase activity, and to confirm whether fungal infection may affect the levels of prostaglandin E1 (PGE1), prostaglandin E2 (PGE2), prostaglandin A1 (PGA1), prostaglandin F2α (PGF2α), 8-iso prostaglandin F2 alpha (8-iso-PGF2α), leukotriene B4 (LTB4), thromboxane A2 (TXA2), thromboxane B2 (TXB2), and 11-dehydrothromboxane B2 (or 11-dehydro-TXB2) in these insects.

## Materials and Methods

### Insects

A culture of the wax moth, *Galleria mellonella* (Lepidoptera: Pyralidae), was maintained in temperature and humidity-controlled chambers (30°C, 70% r.h.) in constant darkness on an artificial diet (Sehnal, 1966). Fully grown larvae were collected before pupation, surface-sterilized and homogenized, and then used as a supplement in the fungal cultures. Five-day-old last instar larvae were used to determine the influence of fungal infection on hemocyte apoptosis, caspase activity, eicosanoid concentration and PLA2 activity.

### Fungus

*Conidiobolus coronatus* (isolate number 3491), originally isolated from *Dendrolaelaps* spp., was obtained from the collection of Prof. Bałazy (Polish Academy of Sciences, Research Center for Agricultural and Forest Environment, Poznań). It was routinely maintained in 90 mm Petri dishes at 20°C under a 12-h L:D photoperiod to stimulate sporulation on Sabouraud agar medium (SAM) with the addition of homogenized *G. mellonella* larvae to a final concentration of 10% wet weight. The sporulation and virulence of the SAM *C. coronatus* cultures was enhanced with the addition of homogenized *G. mellonella* larvae.

### Infection of Insects With *Conidiobolus coronatus*

*Galleria mellonella* larvae (5-day-old last instar) were exposed for 24 h to fully grown and sporulating *C*. *coronatus* colonies. Fifteen individuals were maintained in each Petri dish. A control group was formed of larvae exposed for 24 h to sterile Sabouraud agar medium (Merck). After exposure, the insects were transferred to new, clean Petri dishes with appropriate food [an artificial diet (Sehnal, 1966)] and kept at 20°C for one day. Following this 24-h exposure to the fungus, one group of insects was collected immediately for examination (F24 group) while the rest were left for another 24 h before collection (F48 group).

### Larval Hemolymph Collection

*Galleria mellonella* hemolymph was collected from both control and infected (F24 and F48) larvae. Due to the high mortality of insects (65 ± 5.6% in F24 and 87 ± 4.8% in F48), hemolymph was collected from both living and dying individuals. Before bleeding, the larvae were immersed briefly in 70% (v/v) ethanol to sterilize their surfaces and then washed with distilled water, reducing the contamination of hemolymph samples. Hemolymph was taken from the larvae through an incision made in the last proleg. The hemolymph was prepared in various ways depending on the planned method.

For hemocyte culture, 100 μl of fresh hemolymph collected from ten larvae was suspended in 500 μl of supplemented Grace’s Insect Medium (GIM; Thermo Fisher Scientific) with added gentamycin (10 mg/ml; Thermo Fisher Scientific), amphotericin B (250 μg/ml; Thermo Fisher Scientific) and phenylthiourea (PTU; 0.1 mM; Merck Millipore). Then, it was transferred to a six-channel μ-Slide IV 0.4 (IBIDI)- 100 μl for each channel. The slides were incubated in 30°C for 24 h.

For analysis of apoptosis/necrosis, 100 μl of fresh hemolymph collected from ten larvae was suspended in 100 μl of supplemented GIM with 10 mM EDTA (ethylenediaminetetraacetic acid) and 30 mM sodium citrate. The suspension was then subjected to flow cytometry.

For quantitative measurement of multi caspase activity and caspase-1 activity, 200 μl of fresh hemolymph was collected from twelve larvae and suspended in 100 μl of supplemented GIM.

For eicosanoid detection, 200 μl of fresh hemolymph was collected from 12 larvae and suspended in 100 μl of supplemented GIM. Samples were sonicated (20 kHz, 3 min) for cell lysis. The samples were centrifuged at 10,000 × *g* for 10 min to pellet debris. The supernatants were transferred to a new microcentrifuge tube and stored at –20°C before analysis with ELISA (enzyme-linked immunosorbent assay).

PLA 2 activity was measured in 200 μl of fresh hemolymph collected from 12 larvae. The hemolymph was suspended in 100 μl of Reaction Buffer included in sPLA2 Activity Kit (Enzo Life Sciences).

### Apoptosis/Necrosis in Larval Hemocytes After Fungal Infection

The apoptosis/necrosis test was performed in all samples (controls, F24 and F48) using two methods: fluorescence microscopy and flow cytometry.

Examination using fluorescence microscopy was carried out in cell cultures using the GFP CERTIFIED Apoptosis/Necrosis Detection Kit (Enzo Life Sciences). The kit includes Annexin V, which selectively recognizes the phospholipid phosphatidylserine (PS). Under physiological conditions, PS occurs in the cell membrane from the cytosol side. During the early phase of apoptosis, the PS in the cytosol is moved out of the cell *via* the cell membrane. In this kit, Annexin V was conjugated with green fluorescence protein (GFP). The Necrosis Detection Reagent similar to the red-emitting dye 7-AAD (7-aminoactinomycin D), facilitates late apoptosis and necrosis detection.

The hemolymph taken from control, F24 and F48 larvae was subjected to 24-h incubation at 30°C. Following this, adherent hemocytes were washed twice with PBS (Merck Millipore). A mixture consisting of 5 μl Apoptosis Detection Reagent – Annexin V EnzoGold, 5 μl Necrosis Detection Reagent and 500 μl Binding Buffer was added to μ-Slide, 85 μl for each channel. The samples were protected from light and incubated for 15 min at room temperature, following which, the cells were washed three times with PBS. The cell nuclei were stained with 200 μg/ml Hoechst (Enzo Life Sciences). The hemocytes were observed under an Axio Vert.A1 fluorescence microscope (Zeiss) with Axio Cam ICc 5 (Zeiss) and ZEN 3.2 lite software with Modul Image Analysis (Zeiss). Each test was performed in three independent replicates.

The flow cytometry analysis of apoptosis/necrosis was performed using a Dead Cell Apoptosis Kit with Annexin V FITC and PI (Thermo Fisher Scientific). Hemolymph collected from control and infected larvae was centrifuged at 400 × *g* for 10 min and washed with PBS. The cell pellet was then suspended in the annexin-binding buffer and incubated with 5 μL of FITC annexin V and 1 μl of the 100 μg/ml PI (propidium iodide). Readings were acquired on a CyFlow Cube 8 (Sysmex) and analyzed with FCS Express 7 (*DeNovo* Software). The results are shown in two dot plots, one comparing forward scatter (FSC) with side scatter (SSC) and another comparing PI with Annnexin-FITC.

A positive control was prepared by inducing apoptosis in hemocytes by the administration of staurosporine and actinomycin D and exposing them to UV radiation (as recommended by the manufacturer of the commercial kit used). Apoptosis inducers dissolved in ethanol were added to the hemocytes, to make final concentrations of 1 μM of staurosporine or 10 μg/ml of actinomycin D. In the third positive control variant, the hemocytes were left under a lamp emitting UV radiation for 15 min. All three types of cultures were incubated for 4 h. After this time, an apoptosis/necrosis test was performed according to the procedure described above.

### Activity of Caspases in Larval Hemocytes After Fungal Infection

Caspase activity was measured using the Carboxyfluorescein MultiCaspase Activity Kit (Enzo Life Sciences). This kit contains FAM-VAD-FMK: a carboxyfluorescein derivative of valylalanylaspartic acid fluoromethyl ketone (VAD-FMK), which is a potent inhibitor of caspase activity. The FAM-VAD-FMK reagent enters the cell and covalently binds to the reactive cysteine (Cys 285) on the large subunit of the caspase heterodimer. The VAD multi-caspase substrate allows the detection of caspases 1–9. Following fungal infection, the caspase activity of hemocytes was determined using fluorescence microscopy and fluorometry. Each test was performed in three independent replicates.

For fluorescence microscopy analysis, 24-h cell cultures were used (*viz.* control, F24 and F48). The cells were washed twice with wash buffer (added to kit), then 30X FAM-VAD-FMK solution was added to each slide channel. The mixture was then incubated at 37°C for 1 h in the dark. Necrotic, dead hemocytes were stained using 250 μg/ml propidium iodide (PI), and the cell nuclei were stained with 200 μg/ml Hoechst (Enzo Life Sciences). The hemocytes were observed under an Axio Vert.A1 fluorescence microscope (Zeiss) with Axio Cam ICc 5 (Zeiss) and ZEN 3.2 lite software with Modul Image Analysis (Zeiss).

For quantitative analysis using fluorometry, FAM-VAD-FMK was added to all tested and control hemocyte suspensions in a ratio of 1:30. The samples were incubated at 37°C for 1 h, protected from light. After this time, the samples were washed three times in the washing buffer by centrifugation (5 min, 400 × *g*, room temperature). After the last washing, the pellet was dissolved in 400 μl of PBS. The measurement was performed on black 96-well Assay Plates (DNA Gdańsk) with 100 μl of suspension in each well. The fluorescence intensity of fluorescein (excitation 490 nm, emission 520 nm) was then measured using a Synergy HT Microplate Reader (BioTek).

### Caspase-1 Activity in Hemolymph

Caspase-1 activity was then measured using the Caspase-1 colorimetric assay kit (Enzo Life Sciences). The assay is based on spectrophotometric detection of the chromophore *p*-nitroaniline (pNA) after cleavage from the labeled substrate YVAD-pNA. Light emission from pNA was quantified using a Synergy HT Microplate Reader (BioTek) at 405 nm. The results are presented as the percentage of activity in relation to controls, assumed as 100%.

### Eicosanoid Concentration in Hemolymph

Quantitative eicosanoid analysis was carried out using ELISA tests purchased from Enzo Life Sciences. The following commercial kits were used: PGE1 ELISA kit, PGE2 ELISA kit, PGA1 ELISA kit, PGF2α ELISA kit, Direct 8-iso-PGF2α ELISA kit, LTB4 ELISA kit, TXA2 ELISA kit, TXB2 ELISA kit, 11-dehydro-TXB2 ELISA kit. Each test was performed in four independent replicates according to the manufacturer’s instructions.

### Phospholipase A2 Activity in Hemolymph

The PLA2 activity in the sampled larval hemolymph was detected using the sPLA2 Activity Kit (Enzo Life Sciences). The kit uses a specific substrate for PLA2 that is converted into a sulfhydryl molecule, which is then detected using Ellman’s reagent, DTNB, i.e., 5,5’-dithiobis-(2-nitrobenzoic acid); a positive sample is indicated by a yellow-colored product. The amount of PLA2 in the sample is measured by colorimetry at 405 nm and compared with a standard. The test was performed in three independent replicates according to the manufacturer’s instructions.

### Statistics

The normality of the data was tested using the Kolmogorov–Smirnov test. The *t*-test for independent samples was used to compare the results of the control group and the study group. Pearson’s correlation test was used to find out about the relationship between the concentration of eicosanoids and the activity of caspases. The results were regarded as being statistically significant at *p* ≤ 0.05. STATISTICA 6.1 software (StatSoft Polska) was used for all calculations.

## Results

### Apoptosis and Necrosis in Insect Hemocytes

The *G. mellonella* larvae hemoctytes were tested for apoptosis/necrosis using fluorescence microscopy and flow cytometry.

**FIGURE 1:**
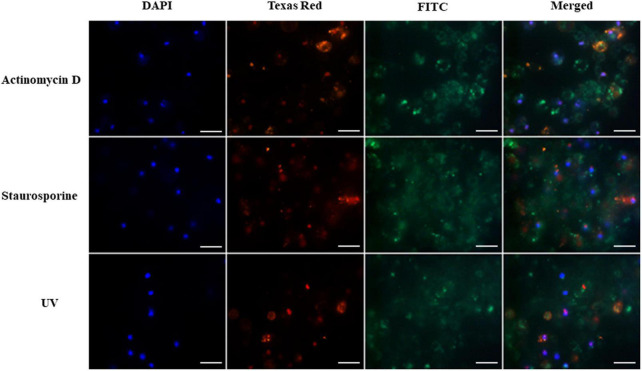
Induction of apoptosis and necrosis in hemocytes of *G. mellonella* larvae by staurosporine, actinomycin D and UV radiation (positive controls). The analysis was performed using the GFP CERTIFIED Apoptosis/Necrosis Detection Kit (Enzo Life Sciences). Texas Red filter, necrosis/late apoptosis detected by 7-AAD; FITC filter, apoptosis detected by Annexin V conjugated with GFP; DAPI filter, cell nuclei; scale bar 25 μm.

**FIGURE 2:**
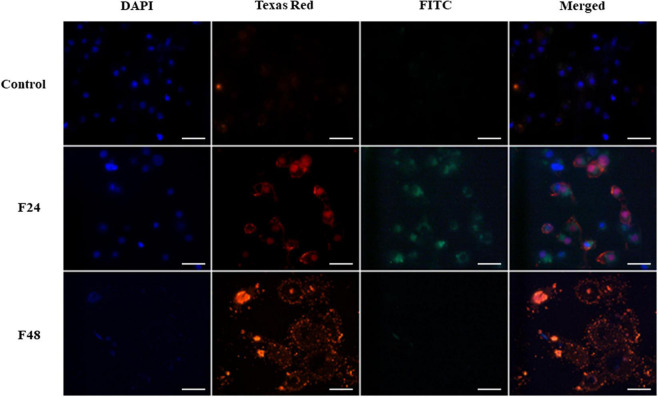
Fungal infection of *G. mellonella* larvae induced apoptosis and necrosis of hemocytes. The analysis was performed using the GFP CERTIFIED Apoptosis/Necrosis Detection Kit (Enzo Life Sciences). Texas Red filter, necrosis/late apoptosis detected by 7-AAD; FITC filter, apoptosis detected by Annexin V conjugated with GFP; DAPI filter, cell nuclei. Control (negative control), non-infected, healthy larvae; F24, larvae sampled immediately after 24-h exposure to *C. coronatus* sporulating colonies; F48, larvae sampled 24 h after 24-h exposure; scale bar 25 μm.

In the fluorescence microscopy, cell nuclei were imaged in the DAPI channel (blue). Hemocytes in late apoptosis or necrosis were indicated by orange fluorescence on the Texas Red channel. Apoptotic cells were stained green and could be seen in the FITC channel. The merged images are given in Figures 1, 2.

Hemocytes treated with actinomycin D, staurosporine and exposed to UV radiation were used as positive controls. The results are shown in Figure 1. The presence of intense green fluorescence on the FITC channel indicated that most of the cells were apoptotic; however, while fluorescence was observed on the Texas Red channel, indicating that actinomycin D, staurosporine and UV radiation caused necrosis in some cells, apoptosis appears to predominate. The cell nuclei also appeared to demonstrate irregular shapes and structures.

A microscope image of *G. mellonella* hemocytes collected from healthy insects (control) and after fungal infection (F24- larvae sampled immediately after 24-h exposure to fungal infection; F48- larvae sampled 24 h after 24-h exposure) is given in Figure 2. In the control, very weak fluorescence was observed on Texas Red and FITC, which indicated that most hemocytes from healthy insects did not demonstrate apoptosis or necrosis. The cell nuclei (DAPI channel) were of correct shape and structure. In the F24 group, intense green and orange fluorescence was observed, indicating the occurrence of both necrosis and apoptosis. Compared to controls, the cell nuclei of some larval hemocytes were enlarged or demonstrated chromatin condensation. In the F48 group, necrosis (or late apoptosis) dominates, as evidenced by intense fluorescence on the Texas Red channel. However, apoptosis was not observed in hemocytes collected from these insects, i.e., no green fluorescence was seen. The cell nuclei were broken down and only their fragments were visible on the DAPI channel.

The fluorescence microscopy images were analyzed in more detail using ZEN Image Analysis Modul software. The Mean Intensity Value of each channel was determined and the gray values (GV) of all measured regions were analyzed: briefly, the RGB (Red, Green, Blue) color image was converted to a gray level image in accordance with the formula (R + G + B)/3 and the corresponding average was then displayed. These findings confirmed the microscope observations. The highest intensity of the FITC channel, indicating the process of apoptosis, was recorded for the positive control (actinomycin D- 4724GV; staurosporine- 4951GV; UV-4584) and F24 (3224GV). In the F48 group, strong evidence of necrosis was observed, as indicated by the high intensity (2304GV) of the TexasRed channels, compared to the lower FITC intensity (124GV). In the negative control sample (hemocytes from healthy insects), both the red and green channels demonstrated low intensity, amounting to respectively 122GV and 98GV.

**FIGURE 3:**
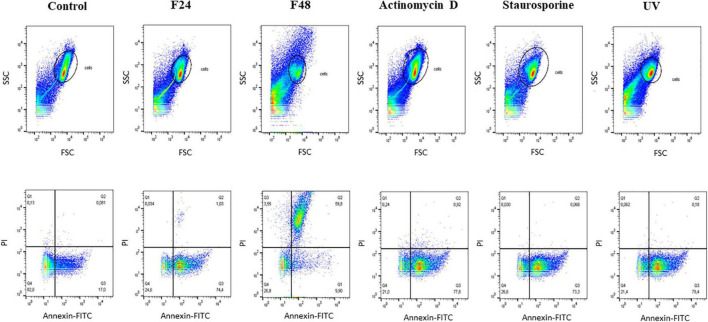
Flow cytometry analysis of apoptosis and necrosis induced in *G. mellonella* hemocytes by *C. coronatus* infection. The analysis was performed using the Dead Cell Apoptosis Kit with Annexin V FITC and PI (Thermo Fisher Scientific). The results are shown as two dot plots comparing forward scatter (FSC) with side scatter (SSC) and PI with Annnexin-FITC. Fungal infection-induced changes were compared to controls, both negative and positive. Control (negative control), non-infected, healthy larvae; F24, larvae sampled immediately after 24-h exposure to *C. coronatus* sporulating colonies; F48, larvae sampled 24 h after 24-h exposure; Actinomycin D, staurosporine and UV- positive controls.

The results obtained using flow cytometry are presented in Figure 3 as dot plots: SSC vs. FSC and PI vs. Annexin FITC. The highest numbers of live cells were observed in the control group (Q4, 82.8%), which also demonstrated few cells in apoptosis (Q3, 17%). Considerable variations in apoptosis and necrosis were found between the two groups of samples collected from infected larvae: while apoptotic cells dominated (Q3, 74.4%), and necrotic cells (Q2) constituted only 1.03% in the F24 group, necrotic cells dominated in the F48 group (Q2, 59.8%) and only 9.9% of the cells were in apoptosis (Q3). Similar percentages of live hemocytes were observed in the two groups: 24.6% in F24 and 26.8% in F48. In F48, many cells were found to have broken down, as indicated by the FSC vs. SSC dot plot and the fluorescence microscopy images. Apoptotic cells (Q3) were found to dominate in the samples incubated with actinomycin D and staurosporine and subjected to UV exposure (positive control samples).

### Caspase Detection and Activity in Hemocytes After Fungal Infection

**FIGURE 4:**
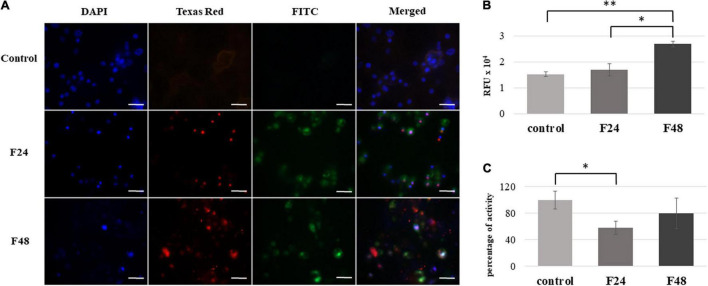
Effect of *C. coronatus* infection on caspase activity in *G. mellonella* hemocytes. The multicaspase analysis was performed using the Carboxyfluorescein MultiCaspase Activity Kit (Enzo Life Science): **(A)** Fluorescence microscopy detection. Texas Red filter, propidium iodide (PI); FITC filter, carboxyfluorescein (FAM); DAPI filter, cell nuclei; scale bar 25 μm. **(B)** Quantitative analysis using fluorometry **(C)** Caspase-1 activity measurements were performed using the Caspase-1 colorimetric assay kit (Enzo Life Science). The results are presented as a percentage of activity in relation to controls, assumed as 100%. Data are presented as means and standard deviations; RFU, Relative Fluorescence Unit. ^∗^*p* ≤ 0.005, ^∗∗^*p* < 0.0001 (Student’s *t*-test, *p* ≤ 0.05). Control (negative control), non-infected, healthy larvae; F24, larvae sampled immediately after 24-h exposure to *C. coronatus* sporulating colonies; F48, larvae sampled 24 h after 24-h exposure.

Multi-caspase activity in *G. mellonella* larval hemocytes after *C. coronatus* infection is shown in Figure 4 as fluorescence microscopy images (Figure 4A) and fluorometry measurements (Figure 4B).

The photographic documentation indicates that the infection of wax moth larvae with entomopathogenic fungus is associated with caspase activation in the hemocytes. Strong green fluorescence was observed on the FITC channel in both the F24 and F48 samples. This indicates a high concentration of FAM in the cell, which is caused by the destruction of the FAM-VAD-FMK complex and the attachment of the inhibitor to active proteins. In both groups, the cell nuclei fluoresced red, which indicates the presence of late apoptosis or necrosis in the hemocytes; however, these processes were more visible in group F48. In addition, the cell nuclei appeared abnormal, as indicated on the DAPI channel. In contrast, only slight autofluorescence was found on the FITC and Texas Red channels in the control group (healthy insects).

Fluorometry indicated significantly greater multi-caspase activity in the F48 group compared to controls (*t* = –16.1, df = 4, *p* < 0.0001), and a statistically significant difference was noted between F24 and F48 (*t* = –7.56, df = 4, *p* = 0.002). The values are presented as Relative Fluorescence Units (RFU).

Caspase-1 activity in *G. mellonella* hemolymph was measured. Spectrofluorimetric data are presented as percentage of activity in relation to control values, assumed as 100% (Figure 4C). In the F24 group, a statistically significant (*t* = 3.14, df = 4, *p* = 0.005) decrease in caspase-1 activity was noted (58 ± 9.7%) compared to control values; however, this activity then increased to 79.6 ± 23.2% over the following 24 h, i.e., the time where the larvae were not exposed to the fungus (group F48).

### Quantitative Measurement of Eicosanoid Concentration in Hemolymph

**FIGURE 5:**
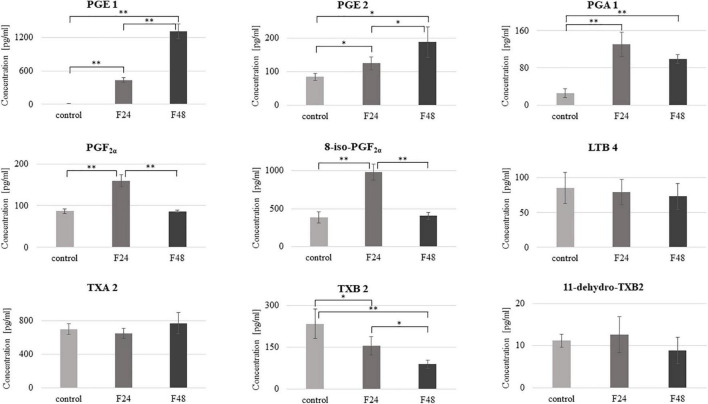
Concentration of selected eicosanoids in the hemolymph of *G. mellonella* larvae after infection with *C. coronatus.* The measurements were performed using ELISA tests. Data are presented as means and standard deviations. ^∗^*p* ≤ 0.05, ^∗∗^*p* < 0.001 (Student’s *t*-test, *p* ≤ 0.05). Control, non-infected, healthy larvae; F24, larvae sampled immediately after 24-h exposure to *C. coronatus* sporulating colonies; F48, larvae sampled 24 h after 24-h exposure.

In healthy *G. mellonella* larvae (control) and those subjected to fungal infection (F24 and F48), the hemolymph concentrations of prostaglandins (PGE1, PGE2, PGA1, PGF2α, 8-iso-PGF2α), thromboxanes (TXA2, TXB2, 11-dehydro-TXB2) and leukotriene (LTB4) were determined using ELISA tests. The results are summarized in Figure 5.

The predominant eicosanoids in the control and F48 groups was TXA2 (702 pg/ml), while 8-iso-PGF2α predominated in F24 (981 pg/ml) and PGE1 in F48 (1308 pg/ml). In contrast, the lowest hemolymph concentrations of TXB2 were observed in both healthy and infected insects. Fungal infection was found to affect the hemolymph levels of most of the tested eicosanoids apart from LTB4, TXA2 and 11-dehydro-TXB2. PGE1 demonstrated a statistically significant increase in both infected groups (F24 and F48) compared with controls (*t* = –18.6, df = 6, *p* < 0.0001), as did PGA1 (*t* = –7.6, df = 6, *p* < 0.0001) and PGE2 (*t* = –3.6, df = 6, *p* = 0.01 for F24 and *t* = –4.4, df = 6, *p* = 0.004 for F48, respectively). On the other hand, statistically significant increases in PGF2α and 8-iso-PGF2α levels were observed only in the F24 group (*t* = –9.8, df = 6, *p* < 0.0001 and *t* = –9.2, df = 6, *p* < 0.0001, respectively). Compared to F24, the levels of PGE1 (*t* = –12.3, df = 6, *p* < 0.0001) and PGE2 (*t* = –2.6, df = 6, *p* = 0.04) were significantly elevated in F48 while those of PGF2α (*t* = 10.5, df = 6, *p* < 0.0001), 8-iso-PGF2α (*t* = 9.8, df = 6, *p* < 0.0001) and TXB2 (*t* = 3.6, df = 6, *p* = 0.01) were insignificantly lower. Only thromboxane B2 was significantly lower in both F24 and F48 than controls (for F24 *t* = 2.5, df = 6, *p* = 0.04; for F48 *t* = 5.3, df = 6, *p* = 0.001). The exact numerical data of the eicosanoid concentration are given in the Supplementary Table S1.

Pearson’s correlation coefficients were calculated between the eicosanoid concentrations and multicaspase activity or caspase-1 activity (Table 1). A statistically significant negative correlation was found between PGE 2 and multicaspase activity in controls and a positive correlation between LTB4 and caspase-1 activity in group F24.

**TABLE 1:** Pearson’s correlation coefficients (*r*) between the concentration of selected eicosanoids and multicaspase activity or caspase-1 activity.

	Multicaspase activity			Caspase-1 activity		
	Control	F24	F48	Control	F24	F48
PGE 1	0.46	0.84	–0.39	0.19	0.05	–0.40
PGE 2	**–0.96**	0.11	0.17	0.47	–0.84	0.73
PGA 1	0.07	–0.53	–0.62	–0.83	0.84	–0.35
PGF_2α_	–0.07	0.41	0.64	0.85	–0.43	0.35
8-iso-PGF_2α_	–0.48	–0.30	–0.72	0.78	–0.70	0.29
LTB 4	0.12	–0.24	–0.67	–0.41	**0.99**	–0.14
TXA 2	–0.45	0.27	0.31	–0.20	0.76	0.63
TXB 2	0.30	–0.67	–0.16	0.47	–0.61	0.02
11-dehydro-TXB 2	0.02	–0.24	0.08	0.79	–0.30	–0.78

*Between ±0.50 and ±1, strong correlation**;** between ±0.30 and ±0.49, medium correlation**;** below ±0.29, low correlation.*

*Control (negative control), non-infected, healthy larvae; F24, larvae sampled immediately after 24-h exposure to C. coronatus sporulating colonies; F48, larvae sampled 24 h after 24-h exposure.*

*Statistically significant values (p ≤ 0.05) are bold and underlined.*

### Phospholipase A2 Activity in Hemolymph After Fungal Infection

**FIGURE 6:**
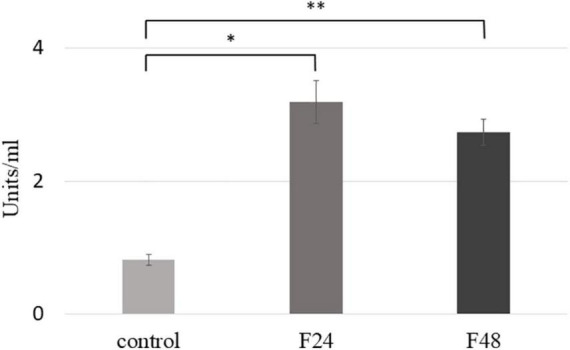
Phospholipase A2 (PLA2) activity in the hemolymph of *G. mellonella* larvae after infection with *C. coronatus.* The measurement was made using sPLA2 Activity Kit (Enzo Life Science). Data are presented as means and standard deviations; ^∗^*p* = 0.0002, ^∗∗^*p* = 0.0001 (Student’s *t*-test, *p* ≤ 0.05). Control, non-infected, healthy larvae; F24, larvae sampled immediately after 24-h exposure to fungal infection; F48, larvae sampled 24 h after 24-h exposure.

Phospholipase A2 activity was measured using the sPLA2 Activity Kit (Enzo Life Science); the results are presented in Figure 6. Significant increase in PLA2 activity was noted 24 h (F24, *t* = –12.3 df = 4, *p* = 0.0002) and 48 h (F48, *t* = –15.5, df = 4, *p* = 0.0001) after infection with *C. coronatus*. A slight, but not significant, decrease in PLA2 activity was observed in the F48 group compared with the F24 group (*p* < 0.05).

## Discussion

Although insects are one of the most diverse groups of animals, comprising more than a million described species and representing more than half of all known living organisms, the nature of apoptosis in this group remains unclear, as most studies examining apoptotic pathways have been limited to the *Drosophila* system (Jacobs et al., 2020; Li et al., 2020). This is a significant topic of research, as apoptosis is known to be an effector mechanism marshal for the pathogen defense in insects. Indeed, our previous research based on the increasingly popular lepidopteran *G. mellonella* model (Sheehan et al., 2018; Piatek et al., 2020) indicated that hemocytes from larvae undergo simultaneous apoptosis and necrosis 24 h after infection with *C. coronatus*, and that this is followed by further cell breakdown and necrosis after another 24 h, resulting in the death of 97% of infected insects (Wronska et al., 2018).

In the present study, the apoptotic cells demonstrated greater chromatin condensation and cell nuclei enlargement compared to untreated controls; this is a classic feature of apoptosis induced by nucleases such as caspase-activated deoxynuclease (CAD), or caused by direct DNA destruction by reactive oxygen species (Wlodkowic et al., 2011). In contrast necrotic hemocytes demonstrated cell nucleus breakdown.

In the Lepidoptera, the best known example of apoptosis is that of insect metamorphosis, where during the larva-pupa transition the larval midgut epithelium of is progressively displaced by a new epithelial layer that grows underneath it; as a result, the larval midgut is pushed into the gut lumen, where it forms the yellow body, a compact mass of cells that subsequently die (Romanelli et al., 2016). However, apoptosis also plays an important role in preventing infection in insects (Eleftherianos et al., 2021). Studies on baculoviral infection suggest that apoptosis can be an extremely powerful response to infection, reducing viral replication, infectivity, and the ability of the virus to spread within the insect host even if successful infection is established. Apoptosis is especially effective when it is combined with other innate antiviral defenses (Clarke and Clem, 2003; Clem, 2005; Nainu et al., 2015). Most literature data on the role of apoptosis in the defense of insects against bacterial infections concern the mechanisms utilized by pathogens to repress the host apoptotic response (Ramphul et al., 2015; Tang et al., 2020). For example, silencing of the locust *inhibitor of apoptosis protein 1* (LmIAP1) gene results in greater direct mortality and increased insect susceptibility to *Metarhizium acridum*, a locust-specific fungal pathogen In turn, *Metarhizium anisopliae infection* has been found to induce fatal *apoptosis* in *Aedes aegypti* larvae; this *process appears to depend on* caspase activity, which is regulated by heat shock protein 70 (HSP 70) and inhibited by protease inhibitors (Butt et al., 2013). Our previous studies with *G. mellonella* larvae infected with *C. coronatus* identified elevated levels of HSP 90, 60, and 27 in insect hemolymph, but not of HSP 70, which suggests that the process of fungus-induced apoptosis is associated with other factors than HSP 70, at least in *G. mellonella* (Wronska and Bogus, 2020).

A central role in apoptosis is played by caspases, evolutionarily conserved cysteine-dependent aspartate-specific proteases whose role in mammals is well known and widely described: initiator caspases (caspases 2, 8, 9, and 10) are activated first in the pro-apoptotic proteolytic cascade, and these in turn activate the effector caspases 3, 6, and 7 (Kesavardhana et al., 2020). However, little information exists about the caspases in Lepidoptera, with only six such proteins and/or their genes being described: Sf-caspase-1 (*S. frugipedia*), Sl-caspase-1 (*S. littoralis*), Hearm caspase-1 (*H. armiger*), Gm-caspase-1 (*G. mellonella*), *Cs-*caspase-1 (*Chilo suppressalis*), *Bm*-*caspase-1* (*Bombyx mori*) (Cooper et al., 2009; Accorsi et al., 2015; Wang et al., 2017). Phylogenetic analyses have found Lepidoptera to possess at least six caspases; of these, Lep-Caspase-1, -2, and -3 are believed to be potential effector caspases, and Lep-Caspase-5 and -6 to be initiators, while the function of Lep-Caspase-4 remains unclear (Courtiade et al., 2011). In contrast, seven caspases have been characterized in *Drosophila melanogaster*: three initiators and four effectors (Hounsell and Fan, 2021).

Our present findings indicate that infection with *C. coronatus* induces caspase activation in the hemocytes of *G. mellonella* larvae, with a statistically significant increase in enzyme activity being observed 48 h after infection, as indicated by fluorescence intensity. The tests used in the present study can detect proteins capable of cleaving the FAM-VAD-FMK complex, resulting in the release of carboxylorescein (FAM). Therefore, it can be assumed that enzymes with similar properties to those of known human caspases are active in *G. mellonella* after fungal infection. Despite this, further studies are needed to demonstrate the role of Gm-caspase-1 in triggering apoptosis in insects infected by fungus. While the apoptotic remodeling of the insect body during metamorphosis is known to be controlled by hormones (Poyraz Tinartas et al., 2021) a number of other relatively unknown factors are also believed to modulate the role of apoptosis in the immune response, including arachidonic acid (AA, C20:4, ω-6) derivatives known as eicosanoids. Our findings confirm that PGE1, PGE2, PGA1, PGF2α and 8-iso-PGF2α production is increased in the hemolymph of *G. mellonella* larvae during fungal infection, while TXB2 production is reduced. In contrast, fungal infection had no effect on LTB4, TXA2 or 11-dehydro-TXB2 levels.

Of all known eicosanoids, those with the best defined role in apoptosis are the prostaglandins (PGs), produced through the COX pathway (Wojcik et al., 2020). Our present findings indicate that fungal infection results in elevated PG levels in insect hemolymph and hemocytes apoptosis; despite this, further studies are needed to confirm the contribution of individual PGs to the fungal-induced apoptosis of hemocytes. Most previous studies on eicosanoids have been restricted to their role in mammals.

An important issue will be to determine whether the contribution of eicosanoids to apoptosis in insects is analogous to that observed in mammals. In the latter case, both the intrinsic and extrinsic pathways appear to be involved in PGE2-induced apoptosis, as suggested by the respective activations of caspase-9 and caspase-8 (Yin et al., 2013). It has been proposed that PGE2 suppresses apoptosis in goat granulosa cells, while PGF2α has the opposite effect (Regan et al., 2018). Similarly, COX-2 mediated production of PGE2 has been shown to promote cell growth and suppress apoptosis in various tumors (Finetti et al., 2020). It is well established that four GPCRs (G-protein coupled receptors) are present on the PGE2 protein and that these mediate the inflammatory activity of the protein. Of these four, the PGE2-EP4 receptor signaling pathway appears to have the greatest influence on the antiapoptotic activity of PGE2 in humans (Hawcroft et al., 2007). Interestingly, while PGE2 acts as an antiapoptotic signal in epithelial cells, it also acts as a pro-apoptotic signal in fibroblasts (Thannickal and Horowitz, 2006).

Cyclopentenone prostaglandins such as PGA1 and PGA2 have been found to inactivate p53-dependent transcription and induce p53-independent apoptotic cell death (Lee et al., 2020). More specifically, PGA1 appears to induce apoptosis in mammalian cells by specifically binding to H- and N-Ras on endomembranes and activating them (Anta et al., 2016).

Another group of proteins that influence apoptosis in both mammals and invertebrates, such as *Caenorhabditis elegans, D. melanogaster and the* silkworm *B. mori*, are the Ras proteins. Together with their structurally related cousins, the Ras proteins are members of the small GTPase family, a superfamily of monomeric GTP binding proteins (G-proteins) (Reiner and Lundquist, 2018). Pinal et al. (2019) suggest that the Ras pathway is able to suppress apoptosis in *Drosophila* when overexpressed; however, no such studies have been performed in Lepidoptera, especially during fungal infection, and this may be an interesting topic for further research.

While it has recently been found that prostaglandin A2 induces apoptosis in three cell lines derived from *Spodoptera frugiperda* (Wang et al., 2021), there remains a general need for further studies on the influence of eicosanoids on the process of apoptosis in insects.

Emerging evidence suggests that leukotrienes (LTs) are also involved in apoptosis in mammals. LTB4 appears to induce elevated intracellular Ca^2+^ concentration, cell polarization and retardation of neutrophil apoptosis (Murray et al., 2003), and the leukotriene B4 plays a key anti-apoptotic role in neutrophils by stimulating reactive oxygen species release *via* NADPH oxidase (Barcellos-de-Souza et al., 2012).

Our present findings do not suggest that despite causing hemocyte apoptosis, fungal infection does not appear to influence LTB4 levels in *G. mellonella*, nor does it affect TXA2 or 11-dehydro-TXB2 concentration; however, it does appear to cause a decrease in TXB2 level in hemolymph. Other authors highlight the fact that thromboxanes appear to play both stimulatory and inhibitory roles in apoptosis in mammals (Huang et al., 2014; Cai et al., 2018; Chueh et al., 2020). Inhibition of thromboxane synthases (TXS) leads to caspase activation, DNA fragmentation, and subsequent cell death, predominantly *via* the mitochondrial pathways (Yin et al., 2013). Eicosanoids are known to mediate various immune responses in insects such as microaggregation, nodule formation, prophenoloxidase (PPO) activity, phagocytosis (Stanley, 2006) and hemocyte migration (Merchant et al., 2008). In *S. exigua*, prostaglandins stimulate serine protease activity, resulting in the lysis of oenocytoids that store PPO in the plasma (Shrestha and Kim, 2008).

Stanley and Kim provide a detailed description of the crosstalk between Toll/Imd immune signaling pathways and eicosanoids, emphasizing that Toll and Imd activate PLA2, resulting in eicosanoid biosynthesis and activity (Stanley and Kim, 2018). In *Tribolium castaneum*, both the Toll and Imd signal pathways induce the expression of specific AMPs, which activate eicosanoid biosynthesis in response to various bacterial infections (Shrestha and Kim, 2010). Eicosanoids mediate immune reactions to bacterial infections in tobacco hornworms *Manduca sexta* (Stanley-Samuelson et al., 1991) and modulate expression of antimicrobial peptides (AMPs) such as lysozyme and cecropin in *Bombyx mori* (Morishima et al., 1997).

Eicosanoids have also been found to insect response to fungal infection. Dean et al. found that eicosanoids mediate cellular defenses to the pathogenic fungus *Metarhizium anisopliae* in hornworms (*Manduca sexta*). Treating hornworms with eicosanoid biosynthesis inhibitors was found to increase mortality of larvae, what can be prevented by co-injections of AA, the PG precursor. The experimentally treated hornworms also produced fewer nodules indicative of fungal infection (Dean et al., 2002). In addition, Park and Kim showed that eicosanoid biosynthesis is activated *via* Toll, but not Imd signal pathway in response to fungal infection in *S. exigua* (Park and Kim, 2012).

Although the effect of eicosanoids on the immune system of wax moths (*Galleria mellonella*) remains unclear during fungal infection, some studies have examined their activity during viral and bacterial infections. These studies have noted that eicosanoids influence phagocytosis and cell spreading in *G. mellonella*; cell spreading is a phase of nodulation and an important element in the immune response to infection (Kim and Stanley, 2021). In addition, cyclooxygenase products, i.e., prostaglandins, appear to mediate nodulation response to viral infection (Buyukguzel et al., 2007), and that eicosanoids may mediate the immune response following injection of entomopathogenic nematodes (*Steinernema carpocapsae* and *Heterorhabditis bacteriophora*) (Yi et al., 2016) into hemocoel. However, our present study is the first to identify changes in the concentrations of hemolymph eicosanoids during fungal infection in *G. mellonella*, which may reflect their effects on the immune system.

In addition to multi-caspase activity, the present study also examines that of caspase-1. It was found that 24-h fungal infection caused a decrease in caspase-1 activity in *G. mellonella* hemolymph, and that this correlated with changes in eicosanoid concentration. Previous studies have suggested that eicosanoids can modulate caspase-1 activity: Zhang et al. (2015) report that eicosanoid biosynthesis inhibitors may simultaneously decrease HSP 20.8 synthesis and increase caspase-1 activity in *Antheraea pernyi* (Lepidoptera). However, the relationship between caspase-1 and eicosanoids is better understood in mammals: active caspase-1, generated by multiple inflammasomes such as NAIP/NLRC4 and NLRP1, has been found to stimulate rapid eicosanoid synthesis by the activation of cytosolic phospholipase A2 (Rathinam and Fitzgerald, 2016).

Phospholipase A2 (PLA2) catalyzes the first step of eicosanoid biosynthesis by hydrolyzing AA from membrane phospholipids at the sn-2 position (Calder, 2020). Our present findings indicate that infection with the entomopathogenic fungus *C. coronatus* results in increased PLA2 activity in *G. mellonella* hemolymph. Similarly, Park and Kim report a functional relationship between the Toll signal pathway and PLA2 activation in response to fungal challenge (*Beauveria bassiana*) in *S. exigua*, another member of the Lepidoptera (Park and Kim, 2012). In addition, PLA2 inhibition substantially reduced phagocytosis of the parasitic protozoan *Typanosoma rangeli* by *Rhodnius prolixus* hemocytes (Figueiredo et al., 2008). Interestingly, PLA2 activity in hemolymph and combined hemocyte/fat body preparations was also elevated in *Tribolium castaneum* following *E. coli* challenge (Shrestha et al., 2010), and infection with the entomopathogenic bacterium *Xenorhabdus nematophila* was found to induce significant immunosuppression of *S. exigua*, in which PLA2 is believed to play a role (Park and Kim, 2000).

Our present findings confirm our hypothesis that fungal infection induces apoptosis of insect hemocytes, and that such infection also stimulates the general activity of caspases in insects. These processes may be correlated with changes in the concentration of eicosanoids in the hemolymph and an increase in the activity of PLA2, an enzyme involved in their synthesis. Thus, it can be assumed that the process of apoptosis in insects is regulated not only by caspases but also by other factors such as eicosanoids or heat shock proteins (HSP). Our findings are intended as an introduction to further work on apoptosis in insects during fungal infection. We did not want to restrict our analysis to known factors influencing apoptosis, such as caspases and prostaglandins, which are already well-described, but to broaden the topic to include the influence of other eicosanoids. Despite being dedicated to the determination of eicosanoids in mammalian material, the ELISA kits used in the study nevertheless provide an indication of the similarities between many processes taking place in insects and vertebrates. Admittedly, however, these kits have a number of limitations, and so in order to thoroughly confirm the presented hypothesis, further research using more advanced techniques are needed, particularly in the field of molecular biology.

## Data Availability Statement

The original contributions presented in the study are included in the article/Supplementary Material, further inquiries can be directed to the corresponding author/s.

## Author Contributions

AW and AK: conceptualization. AW, AK, and MB: methodology. AW, AK, and MK: investigation. AW: writing – original draft and funding acquisition. MB: writing – review and editing and supervision. All authors: contributed to the article and approved the submitted version.

## Conflict of Interest

MB is the President of BIOMIBO. The remaining authors declare that the research was conducted in the absence of any commercial or financial relationships that could be construed as a potential conflict of interest.

## Publisher’s Note

All claims expressed in this article are solely those of the authors and do not necessarily represent those of their affiliated organizations, or those of the publisher, the editors and the reviewers. Any product that may be evaluated in this article, or claim that may be made by its manufacturer, is not guaranteed or endorsed by the publisher.

## Supplementary Material

Supplementary Table S1 Concentration of selected eicosanoids in the hemolymph of *G. mellonella* larvae after infection with *C. coronatus-* row data. The measurements were performed using ELISA tests. Control, non-infected, healthy larvae; F24, larvae sampled immediately after 24-h exposure to *C. coronatus* sporulating colonies; F48, larvae sampled 24 h after 24-h exposure.
